# Multilayered Films Produced by Layer-by-Layer Assembly of Chitosan and Alginate as a Potential Platform for the Formation of Human Adipose-Derived Stem Cell aggregates

**DOI:** 10.3390/polym9090440

**Published:** 2017-09-13

**Authors:** Javad Hatami, Sandra G. Silva, Mariana B. Oliveira, Rui R. Costa, Rui L. Reis, João F. Mano

**Affiliations:** 1 3B’s Research Group, Biomaterials, Biodegradables and Biomimetics, University of Minho, Headquarters of the European Institute of Excellence of Tissue Engineering and Regenerative Medicine, Avepark—Parque de Ciência e Tecnologia, Zona Industrial da Gandra, 4805-017 Barco GMR, Portugal; jhatami@ua.pt (J.H.); sg82silva@gmail.com (S.G.S.); mboliveira@ua.pt (M.B.O.); rui.costa@dep.uminho.pt (R.R.C.); rgreis@dep.uminho.pt (R.L.R.); 2 ICVS/3B’s, PT Government Associated Laboratory, Braga/Guimarães, Portugal

**Keywords:** layer by layer assembly, chitosan, alginate, cytotoxicity, multilayered film, adhesion, 3D culture, spheroid

## Abstract

The construction of multilayered films with tunable properties could offer new routes to produce biomaterials as a platform for 3D cell cultivation. In this study, multilayered films produced with five bilayers of chitosan and alginate (CHT/ALG) were built using water-soluble modified mesyl and tosyl–CHT via layer-by-layer (LbL) self-assembly. NMR results demonstrated the presences of mesyl (2.83 ppm) and tosyl groups (2.39, 7.37 and 7.70 ppm) in the chemical structure of modified chitosans. The buildup of multilayered films was monitored by quartz-crystal-microbalance (QCM-D) and film thickness was estimated using the Voigt-based viscoelastic model. QCM-D results demonstrated that CHT/ALG films constructed using mesyl or tosyl modifications (mCHT/ALG) were significantly thinner in comparison to the CHT/ALG films constructed with unmodified chitosan (*p* < 0.05). Adhesion analysis demonstrated that human adipose stem cells (hASCs) did not adhere to the mCHT/ALG multilayered films and formed aggregates with sizes between ca. 100–200 µm. In vitro studies on cell metabolic activity and live/dead staining suggested that mCHT/ALG multilayered films are nontoxic toward hACSs. Multilayered films produced via LbL assembly of ALG and off-the-shelf, water-soluble modified chitosans could be used as a scaffold for the 3D aggregates formation of hASCs in vitro.

## 1. Introduction

Mesenchymal stem cells (MSCs), which are capable of self-renewal and multilineage differentiation, are becoming increasingly important for the development of cell therapeutics in regenerative medicine. Adipose-derived stem cell (ASC) represents a very attractive cell type of MSCs that are easily accessible, abundant and rich source of adult stem cells [1]. ASCs are able to self-replicate and can differentiate into osteogenic, adipogenic, and chondrogenic lineages under specific conditions [2]. These potentials, along with their easy accessibility, made ACS a good candidate for many cell-based therapies. The stem cell niche and its microenvironment have important effects on the function and biology of stem cells [3]. For example, the cellular phenotype and biological response of cells are different in a monolayer and 3D culture [4]. Approaches providing a 3D culture environment are becoming popular for cell cultures because they mimic in vivo condition. It was demonstrated that aggregates of bone-marrow-derived MSCs could have therapeutic potential [5,6]. ASCs could represent an ideal candidate for a 3D culture of MSCs because of their abundant autologous cells and the ease of their access. Different techniques, such as non-adherent culture condition, nutrient deprivation, air–liquid surface, spinner flask and hanging drop, have been used to form 3D spheroids [4,6,7].

The layer-by-layer (LbL) deposition of materials by electrostatic assembly has often been used to modify the surface of several materials [8,9,10]. This technique has seen application in the construction of nanostructured and easily tailorable two-dimensional and three-dimensional self-standing structures [11]. Due to its compatibility with the construction of highly organized materials and its high versatility, the LbL deposition method was used for the building of new biomaterials and has seen promising applications in biological field [11,12].

Developing a natural-based multilayer film with tunable size and properties has many applications in the healthcare field [11]. A wide plethora of synthetic and natural origin polyelectrolytes were used as building blocks for LbL construction [13,14,15]. The use of synthetic polymers allows precise control of the polymers’ physicochemical properties, such as molecular weight, while working with low batch-to-batch variation. Moreover, the properties of the polymers may be controlled to withstand a large range of processing parameters, including ionic strength and pH values of assembly, and their chemical modification is usually easily achievable. The main reason for the great potential shown by natural-origin polymers in the biomedical field is their chemical similarity with the native animal extracellular matrix (ECM) [16,17]. Moreover, a great part of such polymers shows a biodegradable behavior and, importantly for electrostatic-driven LbL assembly, most of them are ionizable (i.e., they are polyelectrolytes) [18].

Polysaccharides are an interesting type of polymers found in nature. They often allow high degrees of hydration, are biocompatible, and are often biodegradable [15,16,19,20]. Polysaccharides extracted from marine sources are a particular case of these polymers [16,21], which have seen application in the construction of LbL films. Alginate (ALG), chitosan (CHT), chondroitin sulfate and carrageenans are examples of polysaccharides that have found an application in the construction of nanostructured coatings in two-dimensional materials, self-standing micrometric membranes and three-dimensional structures as hollow tubes, capsules for drug release or cell encapsulation, and scaffolds [22,23,24,25,26]. This array of works supported the wide applicability of such polyelectrolytes, and enhanced their added-value, by incorporating these materials in high-end technological approaches. 

ALG and CHT are two marine-origin polysaccharides with high applicability in the development of biomaterials. ALG is extracted from brown algae and regularly used for cell encapsulation as it is well-known for its lack of cell toxicity [27,28]. CHT is a cationic polyelectrolyte which may be extracted from different sources including shells of crustaceous. Its popularity in the biomedical field is related with its immunological, antibacterial and wound healing properties [29]. Moreover, CHT shares structural similarities with many Glycosaminoglycans (GAGs) present in native ECM [30]. The chitosan/alginate (CHT/ALG) polyelectrolyte pair has been used to construct LbL-based coatings on several materials [31] and a wide plethora of self-standing structures [11,32,33]. The nanostructured multilayered films obtained from the LbL assembly of CHT/ALG polymers are well-characterized regarding thickness variations, permeability to gases and glucose, mechanical properties and cellular response [23]. Importantly, the properties of this system can be modulated by varying LbL deposition conditions including pH, during deposition, and ionic strength. The films may also be post-processed by the chemical or ionic crosslinking of both components, which affects some system’s properties, including their mechanical performance and ability to sustain cell adhesion [23,24].

The application of multilayered films using LbL deposition of CHT/ALG in biological filed is limited because it requires the use of acidic pH values due to the insolubility of CHT in water, at the neutral pH. Martins et al. [34] suggested the use of a water-soluble, commercially available chloride-salt chitosan to prepare CHT/ALG films via LbL assembly in water-soluble conditions, at a neutral pH in order to extend the application of CHT/ALG film in biological fields. Although that work opened up the possibility of processing CHT/ALG films in water-soluble conditions, the modification of CHT with an inorganic salt limits the use of characterization methods, such as NMR, for the full characterization of the modified chitosan. As an example, the presence of the pick of inorganic salt of CHT in NMR spectra interferes with the detection of the degree of substitution of CHT.

In the current work, we suggest the use of multilayered films produced by LbL assembly of mCHT/ALG for spheroid formation of hACSs. Therefore, two chitosan derivatives, prepared by chemoselective functionalizing as an amino group of parent chitosans, were used as cationic polyelectrolyte for LbL construction of multilayered films. Two different synthetized modified chitosans (mCHT) with mesyl and tosyl organic salts were obtained. These modifications conferred chitosan with water and organic solvent (e.g., dimethyl sulfoxide) solubility [35]. Moreover, the use of organic medium resulted in a well-controlled and regioselective modification leading to homogenous products that the degree of modification of CHT could be monitored by NMR. We hypothesized that the modification of chitosan with organic salts, due to their chemistry or size, could tailor the properties of the LbL films. This strategy would allow working with off-the-shelf water-soluble chitosans that could be selected prior to the film deposition according to the desired properties of the film, such as its thickness and viscoelasticity. Though chemoselective functionalizing of chitosan was reported previously for different purposes [36,37], the current work demonstrated the possibility to construct LbL multilayer films and to tailor its thickness and viscoelastic properties using water-soluble modified chitosan with organic salt and alginate polymers for the first time, to the knowledge of the authors, as a supporting scaffold for the 3D culture and aggregate production of hASCs. This approach could be interesting for the immediate adaptation of the properties of the films by maintaining their processing conditions, while simply adjusting the type of chitosan used for their construction, in a user-friendly approach.

## 2. Materials and Methods

### 2.1. Chitosan Purification, Modification and Analysis by ^1^H NMR

CHT of medium molecular weight (MMW–CHT, *M*_W_ = 190−310 kDa, 75–85% degree of deacetylation, viscosity 200–800 cP) was purchased from Sigma-Aldrich (St. Louis, MO, USA). CHT was purified by a sequential filtration and precipitation steps in distilled water, sodium hydroxide and ethanol, as described previously [38], followed by freeze-drying. It was then grounded and stored as a powder until further usage.

**Scheme 1:**
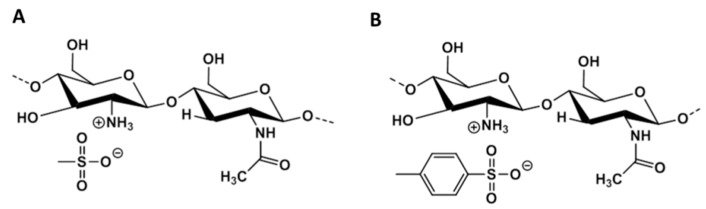
Chemical structure of mesyl–chitosan (CHT) (**A**) and tosyl–CHT (**B**).

Chitosan derivatives were obtained by chemoselective functionalizing as an amino group of parent chitosans. In this work, two organic acids were used to modify the chitosan according to a previously reported method [37]. The result of chitosan modification with these organic acids, methanosulfonic and *p*-toluenosulfonic acids, were mesyl–CHT and tosyl–CHT, respectively (Scheme 1). Briefly, methanosulfonic acid or *p*-tolunesulfonic acid monohydrate was added (1:1), drop-by-drop at 10 °C, to a suspension of pre-purified MMW–CHT until clear homogeneous solutions were obtained. The solutions were then stirred for 2–4 h (10 °C) then washed several times with acetone, to precipitate the salts, and diethyl ether and then, dried in a vacuum oven at 25 °C for 8 h. Before use, the materials were dissolved in distilled water and freeze-dried. The corresponding salts (mesylates and tosylates), which derived from organic acid, was detected by ^1^H NMR (Bruker Avance III operating at 400 MHz, Billerica, MA, USA) through the peaks of the organic anion. To perform NMR analysis, the samples were prepared in D_2_O in the concentration range of 10–15 mg/mL. All the reagents and solvents (p.a. quality) used in the preparation of mCHTs were purchased from Sigma.

### 2.2. Construction and Characterization of mCHT/ALG Multilayered Films by Quartz-Crystal Microbalance

Alginic acid sodium salt from brown algae (Sigma-Aldrich, St. Louis, MO, USA ref. 71238) was dissolved in a solution of NaCl (0.15 M, Sigma) to obtain a final 0.1% (*w*/*v*) alginate solution. Solutions of mesyl–CHT and tosyl–CHT (all at 0.1% *w*/*v*) were obtained by dissolving the mesyl–CHT and tosyl–CHT in a solution of NaCl (0.15 M). A solution of unmodified chitosan (here, normal–CHT) at a concentration of 0.1% (*w*/*v*) was obtained by dissolving normal–CHT in 1% (*v*/*v*) acidic acid (Sigma) followed by the addition of NaCl to a final concentration of 0.15 M. The pH of all solutions was then adjusted to 5.5 using 2 M NaOH and 1% (*v*/*v*) acetic acid. Laser doppler electrophoresis is often used to measure the magnitude of the charge of colloidal suspensions, but is also appropriate to determine the polycationic and polyanionic behavior of solubilized polymeric materials. Therefore, the zeta (ζ)-potential values of solutions were measured using Nano-ZS (Malvern, Worcestershire, UK), at 25 °C.

Deposition of CHT/ALG multilayers on the gold-coated crystals was monitored by a Q-Sense E4 quartz-crystal microbalance (Q-Sense AB) with dissipation monitoring system [39]. Briefly, AT-cut quartz crystal was excited at 5, 25, 35 and 45 MHz (fundamental frequency, 5th, 7th, and 9th overtones, respectively). The initial crystal cleaning was performed in an ultrasound bath at 30 °C followed by immersion in acetone, ethanol and isopropanol. Starting with CHT solution, deposition of polyelectrolytes took place at a constant flow rate of 50 mL/min, 25 °C and pH 5.5 for 22 min. Following the deposition of each polyelectrolyte, a rinsing step with NaCl (0.15 M) at pH 5.5 for 12 min was performed. Changes in frequency (Δ*F*) and dissipation (Δ*D*) were monitored in real-time.

### 2.3. Estimation of the Film Thickness and Properties

The film thickness was estimated using the Voigt-based viscoelastic model [40], integrated in QTools (version 3.1.25.604) provided by Q-Sense (Biolin Scientific, Gothenburg, Sweden). The model is represented by Equations (1) and (2).

(1) $$\Delta F\approx -\frac{1}{2\pi \rho_{0}h_{0}}\{\frac{\eta_{3}}{\delta_{3}}+\sum_{j=k}[h_{j}\rho_{j}\omega -2h_{j}{\left(\frac{\eta_{3}}{\delta_{3}}\right)}^{2}\frac{\eta_{j}\omega^{2}}{\mu_{j}{}^{2}+\omega^{2}\eta_{j}{}^{2}}]\}$$

(2) $$\Delta D\approx \frac{1}{2\pi f\rho_{0}h_{0}}\{\frac{\eta_{3}}{\delta_{3}}+\sum_{j=k}[2h_{j}{\left(\frac{\eta_{3}}{\delta_{3}}\right)}^{2}\frac{\mu_{j}\omega }{\mu_{j}{}^{2}+\omega^{2}\eta_{j}{}^{2}}]\}$$

In Equations (1) and (2), *ρ*_0_ and *h*_0_ are the density and thickness of the quartz crystal, *k* represents total number of thin viscoelastic layers, *η*_3_ is the viscosity of the bulk liquid, *δ*_3_ is the viscous penetration depth of the shear wave in the bulk liquid, *ρ*_3_ is the density of liquid, *μ* is the elastic shear modulus of an over layer, and *ω* is the angular frequency of the oscillation. Estimations were made considering a fixed solvent viscosity of 0.001 Pa (the same as for water) and film density of 1200 kg/m^3^. The density of solvent was changed, by trial and error, between 1000 and 1015 kg/m^3^ until the total error, *χ*^2^, was minimized. Three overtones (5th, 7th and 9th) were used for the calculations. 

### 2.4. Human Adipose Stem Cell (hASC) Culture

To evaluate the biological performance of CHT/ALG multilayered films, cell culture studies were performed using hASCs. Human abdominal subcutaneous adipose tissue samples were obtained after informed consent from patients undergoing lipoaspiration procedure. The retrieval and transportation of the samples has been performed under a valid cooperation protocol established on 12 February 2007 by the 3B’s research group and the Hospital da Prelada (Porto, Portugal), which is approved by the ethical committees of both institutions, and does not have an associated number. This protocol contains all guidelines to retrieve, transport and discard biological samples in accordance with National and European recommendations, which include the signature of an Informed consent form and a standard anonymization procedure for all samples. Isolation and process of samples were carried out within 24 h after surgical procedure according to a protocol previously established [41]. Isolated cells were cultured under basal condition, using minimum essential alpha medium (α-MEM, Sigma), supplemented with 10% (*v*/*v*) fetal bovine serum (FBS, ThermoFisher Scientific, Waltham, MA, USA) and 1% (*v*/*v*) penicillin-streptomycin, until maximum passages of three. Cell culture medium was changed 48 h after initial plating and every 3 days thereafter.

### 2.5. Preparation of Multilayered Films for Cell Culture Studies

Glass surfaces (1.0 cm^2^) were prepared from coverslips (Fisher Scientific) as substrates to build normal, mesyl and tosyl–CHT/ALG multilayered films. Glass surfaces were kept inside six-well plates (Falcon, Corning, NY, USA) and covered by solutions of CHTs and ALG (all 0.1% in aqueous solution with NaCl 0.15 M). LbL assembly started with CHTs solution deposition and followed by ALG solution deposition (each layer with 22 min deposition time), in a sequential manner with intermediate washing step (10 min) with NaCl (0.15 M) to prepare five bilayers of films.

To study the cytotoxicity and effect of the direct contact of mCHT layer with hASCs, an additional layer of CHTs was deposited on top of five bilayers of CHT/ALG films via LbL technique. Samples were sterilized for subsequent cell culture using exposure to UV (1 h).

### 2.6. Cytotoxicity and Viability Analysis

Glass substrates coated with mesyl, tosyl and normal–CHT/ALG multilayered films, prepared according to the previous section, were used for viability and cytotoxicity testing according to ISO 10993-5 guidelines similar to the method described elsewhere [42]. Samples were incubated in triplicate in 2 ml α-MEM with 10% (*v*/*v*) FBS and 1% (*v*/*v*) penicillin-streptomycin in six-well plates at 37 °C, 5% CO_2_, and fully humidified air for 3 days. The resulting mediums, enriched in lixiviates potentially released from the multilayer films, was used for cytotoxicty and viability studies. Therefore, hASCs with an initial density of 8 × 10^4^ cells/cm^2^ was cultured in resulting medium, in 24-well plates, for 3 days. Metabolic activity of cultured cells was determined using a MTS Cell Proliferation Assay Kit (Abcam, Cambridge, UK) at three time points (days 1, 2 and 3). The results were normalized to the negative control (fresh α-MEM with 10% (*v*/*v*) FBS and 1% (*v*/*v*) penicillin–streptomycin) for cytotoxicity analysis and compared to the positive control medium pre-incubated with latex.

Cell viability was assessed by incubating hACSs, pre-cultured in 24-well plates with resulting mediums for 3 days, with a live/dead assay at three time points (day 1, 2 and 3). In brief, the hASCs were incubated for 15 min with 150 μL of calcein AM (Invitrogen, Carlsbad, CA, USA) solution (2 μL calcein/mL DMEM without phenol red) and 150 μL of propidium iodide (PI, Invitrogen, Carlsbad, CA, USA) working solution (2 μL PI stock solution, 1 mg/mL in distilled water) and finally rinsed with PBS. The cells were then immediately visualized in the dark by fluorescence microscopy (Axio Imager Z1m, Zeiss, Oberkochen, Germany).

### 2.7. Cell Adhesion Studies on mCHT/ALG Multilayered Film

Cell morphology and adhesion were assessed according to the protocol previously reported [43]. Briefly, a cell suspension of hASCs (10^6^ cells/mL) was prepared using Tryple Express (ThermoScientific). Cell suspensions (passage 3–5) with the density of 2.0 × 10^4^ cell/cm^2^ were cultured on top of glass surfaces pre-coated with five bilayers of CHT/ALG films using α-MEM supplemented with 10% (*v*/*v*) FBS and 1% (*v*/*v*) penicillin-streptomycin in humidified atmosphere (37 °C, 5% CO_2_). After 24 h, culture medium was removed, samples were washed with PBS and incubated with 10% of formalin at room temperature (RT) for 1 h. Samples were washed with PBS and incubated with 0.1% Triton X for 5 min at RT to permeabilize cells. Samples were washed again with PBS and solutions of 4,6-Diaminidino-2-phenylindole-dilactate (DAPI, 20 mg/mL, Sigma-Aldrich) and phalloidin tetramethylrhodamine B isothiocyanate dyes (phalloidin, 10 mg/mL, Sigma-Aldrich) were added to the samples to stain the nuclei and cytoskeleton F-actin of the hASCs, respectively. After 1 h at RT and protected from light, cells were washed three times with PBS and immediately visualized in the dark condition by reflected light fluorescence microscopy (Axio Imager Z1m, Zeisss, Oberkochen, Germany) to assess cellular adhesion, and morphology.

### 2.8. Statistical Analysis

Statistical analysis was performed by use of IBM SPSS statistics (v. 20, Chicago, IL, USA) with the use of Mann–Whitney U analysis. Differences in results were considered statistically significant at a value of *p* < 0.05. Results are presented as a mean ± standard deviation (SD).

## 3. Results and Discussion

### 3.1. Synthesis and Characterization of the Mesyl–CHT and Tosyl–CHT

**Figure 1:**
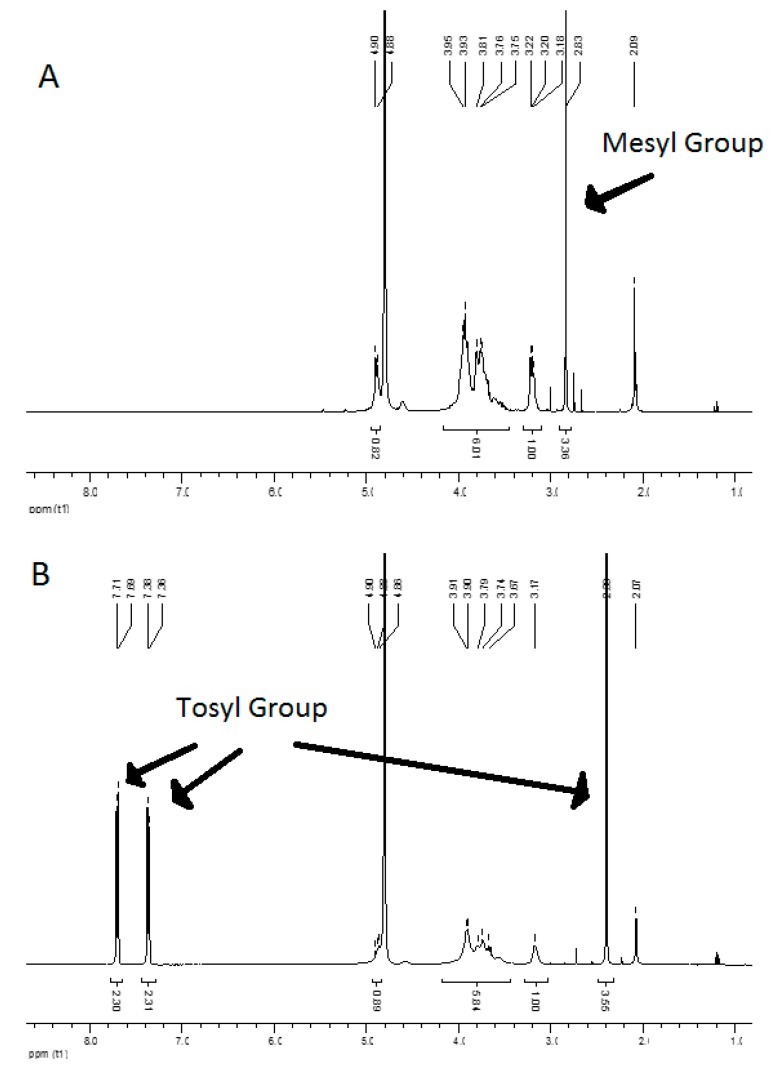
NMR characterization of mesyl–CHT (**A**) and tosyl–CHT (**B**). Peaks of mesyl (2.83 ppm) and tosyl groups (2.39, 7.37 and 7.70 ppm) are highlighted by arrows.

The NMR characterization of mesyl–CHT polymer demonstrated that a peak corresponding to the mesyl group appears at 2.83 ppm as a singlet (Figure 1A) which is in line with results previously described [37]. In the spectrum of the tosylated deriavative, the peaks of the aromatic ring are present at 2.39, 7.37 and 7.70 ppm (Figure 1B). These results confirm chemoselective modification of chitosan with mesyl and tosyl groups. The results also demonstrated that water solubility can be obtained by modification of chitosan with mesyl and tosyl groups.

Since CHT and ALG polyelectrolytes are only partially charged at moderate pH near their p*K*_a_s, working pH and ionic strength are expected to influence film growth and properties. In this work, the pH of CHT was adjusted to 5.5, below its p*K*_a_ value of 6.5 [44], to ensure its performance as a polycation. ζ-potential values show the cationic nature of normal and modified CHTs, as well as the anionic nature of ALG (Table 1) in aqueous solution with 0.15 M NaCl (pH = 5.5). This ensures the existence of electrostatic interactions between CHTs and ALG in order to build LbL films. The charge sign is the same for all chitosans, thus the modification does not change the polycationic character of this polysaccharide. Nonetheless, the results obtained herein provide a qualitative indication that the magnitude of the electrostatic density among normal and modified chitosans was not affected pronouncedly (Table 1).

### 3.2. LbL Build-Up of mCHT/ALG Multilayered Films at pH = 5.5

mCHT and ALG were deposited in a sequential way to build multilayered films. Cationic solutions of mCHTs were deposited on the surface of a gold-coated quartz crystal (negatively charged) and subsequently anionic solution of ALG was deposited in a similar way, with an intermediate washing step using 0.15 M NaCl buffer. Electrostatic interactions between cationic–anionic polyelectrolytes ensures the LbL buildup of multilayered films.

The LbL assembly of the CHT and ALG was monitored by a quartz microbalance with dissipation monitoring (QCM-D). QCM-D analysis proved the effective interaction between the two polyelectrolytes, chitosans and alginate, at pH 5.5. Therefore, it can be said that the assembly of mCHT, and normal-CHT with ALG resulted in the construction of multilayered polymeric films. Alginate, as a polyanion, interacts with the positively charged chitosan. It is assumed that the carboxylate moieties on alginate ionically interact with the protonated amine groups on chitosan to form a matrix.

**Figure 2:**
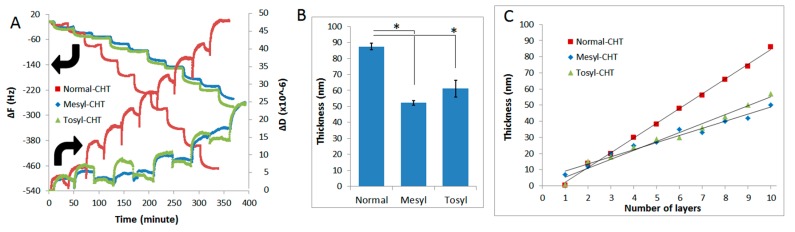
Representative quartz microbalance with dissipation monitoring (QCM-D) acquisition graphics depicting 5th overtone variations of frequency (descending curves) and dissipation (ascending curves) during construction of the multilayered films using normal–CHT/alginate (ALG) (normal), mesyl–CHT/ALG (mesyl) and tosyl–CHT/ALG (tosyl) (**A**); Thickness of films after buildup of 5 bilayers was estimated using Voigt-based viscoelastic model (Average ± SD; * *p* < 0.05) (**B**); Representative cumulative thickness evolution of the multilayer films as a function of the number of deposition layers. Lines represent linear trend lines with *R*^2^ = 0.99 (normal-CHT), *R*^2^ = 0.97 (tosyl–CHT) and *R*^2^ = 0.97 (mesyl–CHT) (**C**).

The frequency of QCM-D crystal decreases by the deposition of a thin film. The decrease in frequency (Δ*F*) is proportional to the mass of the film when the film is thin and rigid. However, this relation is not valid once a soft film is constructed as a result of the deposition of a polymeric material. In this situation, the energy stored in each vibrational cycle is lost and changes in dissipation (Δ*D*) represent a typical viscoelastic behavior. Frequency (∆*F*) and dissipation (∆*D*) variations normalized to the 5th overtone during the construction of 5 bilayers are shown in Figure 2A. The decrease in frequency observed after the adsorption of each successive layers of polyelectrolytes suggests that there was a gradual growth of the polymeric films, using normal and modified chitosans. In general, dissipation values increased with time, revealing that the films are not rigid and can dissipate energy, evidencing their viscoelastic behavior (Figure 2A). ∆*D* provides evidence about film’s viscoelastic properties in which deposition of a soft component often leads to an increase of the dissipation values due to energy loss from the crystal’s oscillation, whereas smaller dissipation values are obtained for rigid components [45,46]. Using the data acquired from the QCM-D experiments, the thickness of the multilayered films was estimated using the Voigt-based viscoelastic model (Figure 2B). The assembly of CHT and ALG followed a linear regime growth (Figure 2C), resulting in films with thickness of 87.4 ± 5.3, 52.3 ± 2.1 and 61.2 ± 1.4 nm for normal, mesyl and tosyl–CHT/ALG multilayers, respectively, after the construction of 5 bilayers. Statistical analysis demonstrated a significance difference between the thicknesses of normal–CHT/ALG and mCHT/ALG films (*p* < 0.05). Thinner mCHT/ALG films obtained by LbL process might be related to the presence of organic salts, mesylate and tosylate, in the chemical structure of mCHTs. The potential to make ionic interactions and form a polyelectrolyte complex (PEC) are among the important reasons that enabled the development of tailored biomaterials using ALG and CHT. The incorporation of modified chitosan in the LbL structure, with meticulous and selectivity practice, would give us the capability to control the thickness and mechanical properties of multilayered films. The possibility to change the physiochemical properties of CHT–ALG PECs by controlling the degree of association between functional groups provides an opportunity to design complex and tailored biopolymer scaffolds.

### 3.3. Cytotoxicity of mCHT/ALG Multilayered Films

**Figure 3:**
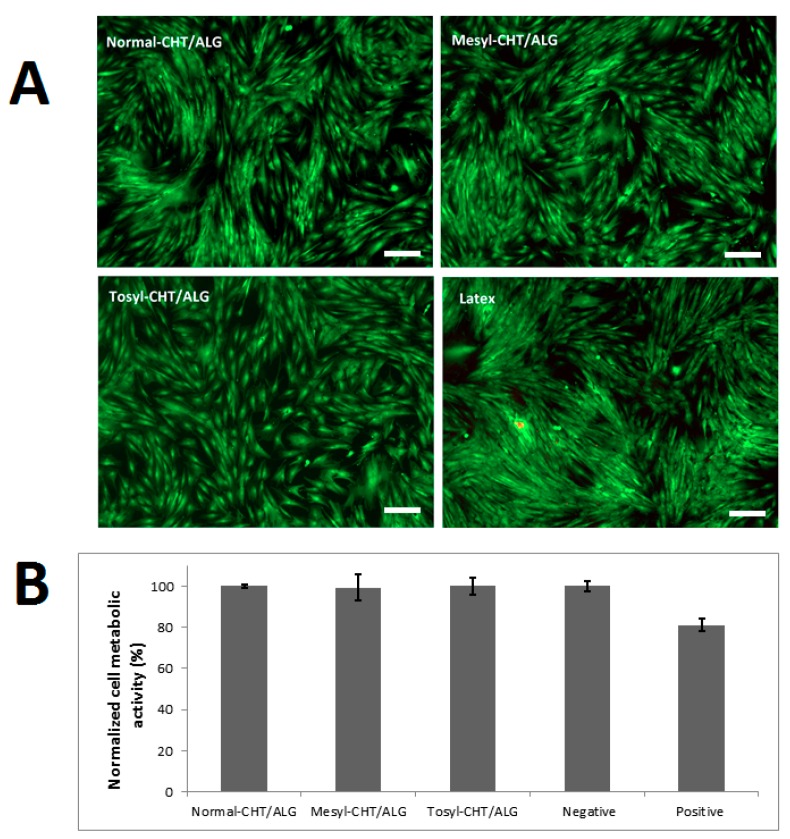
Representative fluorescence images of live (green) and dead (red) hASCs cultured on 24-well plates with the extract of mesyl–CHT/ALG, tosyl–CHT/ALG and normal–CHT/ALG multilayered films and latex. (**A**) (Scale bar = 200 µm); Normalized metabolic activity of hASCs was measured in contact with the extract of CHT/ALG multilayered films as well as negative (24-well plate) and positive (Latex) controls (**B**). Results are presented as average ± SD.

In vitro studies were performed to investigate the cytotoxicity of mCHT/ALG multilayered films using hASCs. Image analysis of live/dead staining by ImageJ (V. 1.51p, Bethesda, MD, USA) displayed a uniform distribution of viable cells (as shown by cells stained with Calcein AM, in green) with viability of 96% ± 4.2% cultured on top of 24-well plate using the resulting culture medium with extract of mCHT/ALG films (Figure 3A). The metabolic activity of hASCs, as a result of incubating with extracts of mesyl–CHT/ALG and tosyl–CHT/ALG multilayered films, was similar to thenormal–CHT/ALG film and in the range of negative control (Figure 3B). However, the pre-incubation of the culture medium with latex resulted in lower metabolic activity and 58% ± 2.3% viability of cells (Figure 3B, positive control). These results further confirmed the cytocompatibility of mCHT/ALG multilayered films for cell culture studies.

### 3.4. Aggregate Formation of hASCs on the mCHT/ALG Multilayered Films

**Figure 4:**
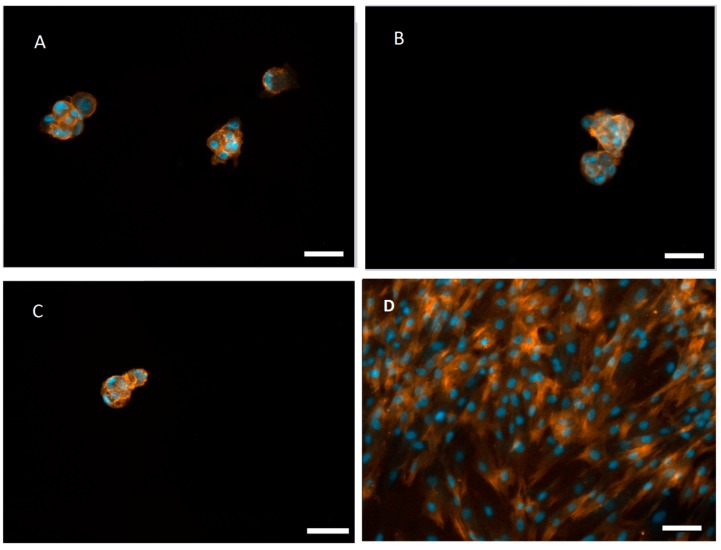
Representative fluorescence images of the hASCs cultured on top of normal–CHT/ALG (**A**); mesyl–CHT/ALG (**B**); tosyl–CHT/ALG (**C**); multilayered films and glass substrate (**D**). Multilayered films included subsequent layers of CHT and ALG with top layer (11th layer) was CHT. Scale bar: 200 µm. Cell nuclei appeared bright blue and F-actin filaments appeared bright red due to DAPI and phalloidin dyes, respectively.

hACSs cultured on normal and mCHT/ALG films did not show the ability to attach and spread to the multilayered films. They assembled in the form of spheroids with sizes between ca. 100 to 200 µm (Figure 4A–C). Low adhesion of the cells in such non-cytotoxic substrate can be used to stimulate cell aggregation. For example, chitosan films were used to prepare spheroids of melanocytes [47] and keratocytes [48]. In contrast, once cultured on glass substrates, hASCs exhibited common spindle shape morphology (Figure 4D). Cells need anchor points to be adhered to the substrate. Some substrates, such as tissue culture plates, are able to absorb the adhesion-related proteins, such as fibronectin, and this allows the attachment of cells to that specific substrate [49,50]. It was previously reported that the amount of adhesive-related proteins was far less in chitosan compared to tissue culture substrates [51]. This result may be attributed to the monopolar nature of chitosan, which is not able to interact with the bipolar extracellular matrix proteins presented in the culture medium. Our result also corroborates previous findings that non-crosslinked free standing CHT/ALG multilayered membranes could not support the adhesion of L929 cells [23]. One could also envisage using the developed multilayers as reservoirs of bioactive agents that could also extend the applicability of these substrates as supports to control cell behavior [52].

## 4. Conclusions

Chitosan and alginate are two natural-based polymers with proved application in developing biomaterials for tissue engineering and regenerative medicine. In this study, we suggest the use of multilayered film produced by LbL assembly of modified chitosan/alginate as a scaffold to produce 3D aggregates of hASCs. We investigated how the modification of chitosan could affect the mCHT/ALG film properties and the corresponding biological performance. Therefore, two water-soluble cationic derivatives of chitosan, namely mesyl and tosyl chitosans, were synthesized and nanostructured multilayered LbL films were created using ALG as an anionic polyelectrolyte. mCHT/ALG films showed lower thickness compared to the normal–CHT/ALG films. Therefore, modification of chitosan with organic salts provided the ability to tailor the physiochemical properties of the multilayered films. The result demonstrated mCHT/ALG multilayered films could be used as a platform to produce 3D aggregates of hACSs. This approach may offer a new route to prepare off-the-shelf chitosan products which have the advantage of solubility in water at a neutral pH, and may be used for customizing the properties of films without the adjustment of processing parameters. Moreover, one could envisage incorporating biological signals in the multilayer films for a variety of applications, such as controlling the behavior of cells. It would be important to further investigate the effect of multilayer properties and spheroid formation on the differentiation potential and stemness of hACSs. This information is useful to develop tailored biomaterials to regulate the function and behavior of hACSs for varieties of applications in tissue engineering and regenerative medicine.

## Figures and Tables

**Table 1:** ζ-Potential values of CHT (0.1%) and ALG (0.1%) in aqueous solution with 0.15 M NaCl (pH = 5.5).

Polyelectrolyteolyte	ζ-Potentialtial
Normal–CHT	+20 ± 1.3
Mesyl–CHT	+22.3 ± 1.6
Tosyl–CHT	+19.8 ± 1.1
Alginate	−28.6 ± 1.7

## References

[1] Gimble M.J., Guilak F. (2003). Differentiation potential of adipose derived adult stem (ADAS) cells. Curr. Top. Dev. Biol..

[2] Guilak F., Lott K.E., Awad H.A., Cao Q., Hicok K.C., Fermor B., Gimble J.M. (2006). Clonal analysis of the differentiation potential of human adipose-derived adult stem cells. J. Cell Physiol..

[3] Jones L.D., Wagers A.J. (2008). No place like home: Anatomy and function of the stem cell niche. Nat. Rev. Mol. Cell Biol..

[4] Frith J.E., Thomson B., Genever P.G. (2010). Dynamic three-dimensional culture methods enhance mesenchymal stem cell properties and increase therapeutic potential. Tissue Eng. C.

[5] Bartosh T.J., Ylöstalo J.H., Mohammadipoor A., Bazhanov N., Coble K., Claypool K., Lee R.H., Choi H., Prockop D.J. (2010). Aggregation of human mesenchymal stromal cells (MSCs) into 3D spheroids enhances their antiinflammatory properties. Proc. Natl. Acad. Sci. USA.

[6] Wang W., Itaka K., Ohba S., Nishiyama N., Chung U.I., Yamasaki Y., Kataoka K. (2009). 3D spheroid culture system on micropatterned substrates for improved differentiation efficiency of multipotent mesenchymal stem cells. Biomaterials.

[7] Oliveira M.B., Neto A.I., Correia C.R., Rial-Hermida M.I., Alvarez-Lorenzo C., Mano J.F. (2014). Superhydrophobic chips for cell spheroids high-throughput generation and drug screening. ACS Appl. Mater. Interfaces.

[8] Borges J., Mano J.F. (2014). Molecular Interactions Driving the Layer-by-Layer Assembly of Multilayers. Chem. Rev..

[9] Richardson J.J., Björnmalm M., Caruso F. (2015). Technology-driven layer-by-layer assembly of nanofilms. Science.

[10] Xiao F.X., Pagliaro M., Xu Y.J., Liu B. (2016). Layer-by-layer assembly of versatile nanoarchitectures with diverse dimensionality: A new perspective for rational construction of multilayer assemblies. Chem. Soc. Rev..

[11] Costa R.R., Mano J.F. (2014). Polyelectrolyte multilayered assemblies in biomedical technologies. Chem. Soc. Rev..

[12] Oliveira M.B., Hatami J., Mano J.F. (2016). Coating Strategies Using Layer-by-layer Deposition for Cell Encapsulation. Chem. Asian J..

[13] Boudou T., Crouzier T., Ren K., Blin G., Picart C. (2010). Multiple Functionalities of Polyelectrolyte Multilayer Films: New Biomedical Applications. Adv. Mater..

[14] Joseph N., Ahmadiannamini P., Hoogenboom R., Vankelecom I.F. (2014). Layer-by-layer preparation of polyelectrolyte multilayer membranes for separation. Polym. Chem..

[15] Banik B.L., Brown J.L., Laurencin C.T., Deng M. (2014). Chapter 23—Polymeric Biomaterials in Nanomedicine A2—Kumbar, Sangamesh G. Natural and Synthetic Biomedical Polymers.

[16] Mano J.F., Silva G.A., Azevedo H.S., Malafaya P.B., Sousa R.A., Silva S.S., Boesel L.F., Oliveira J.M., Santos T.C., Marques A.P. (2007). Natural origin biodegradable systems in tissue engineering and regenerative medicine: Present status and some moving trends. J. R. Soc. Interface.

[17] Hynes R.O. (2009). The Extracellular Matrix: Not Just Pretty Fibrils. Science.

[18] Finkenstadt V.L. (2005). Natural polysaccharides as electroactive polymers. Appl. Microbiol. Biotechnol..

[19] Cardoso M.J., Costa R.R., Mano J.F. (2016). Marine Origin Polysaccharides in Drug Delivery Systems. Mar. Drugs.

[20] Basu A., Kunduru K.R., Abtew E., Domb A.J. (2015). Polysaccharide-Based Conjugates for Biomedical Applications. Bioconjug. Chem..

[21] Senni K., Pereira J., Gueniche F., Delbarre-Ladrat C., Sinquin C., Ratiskol J., Godeau G., Fischer A.M., Helley D., Colliec-Jouault S. (2011). Marine Polysaccharides: A Source of Bioactive Molecules for Cell Therapy and Tissue Engineering. Mar. Drugs.

[22] Oliveira S.M., Santo V.E., Gomes M.E., Reis R.L., Mano J.F. (2015). Layer-by-layer assembled cell instructive nanocoatings containing platelet lysate. Biomaterials.

[23] Silva J.M., Duarte A.R.C., Caridade S.G., Picart C., Reis R.L., Mano J.F. (2014). Tailored Freestanding Multilayered Membranes Based on Chitosan and Alginate. Biomacromolecules.

[24] Caridade S.G., Monge C., Gilde F., Boudou T., Mano J.F., Picart C. (2013). Free-Standing Polyelectrolyte Membranes Made of Chitosan and Alginate. Biomacromolecules.

[25] Cuomo F., Lopez F., Miguel M.G. (2010). Vesicle-templated layer-by-layer assembly for the production of nanocapsules. Langmuir.

[26] Correia C.R., Pirraco R.P., Cerqueira M.T., Marques A.P., Reis R.L., Mano J.F. (2016). Semipermeable Capsules Wrapping a Multifunctional and Self-regulated Co-culture Microenvironment for Osteogenic Differentiation. Sci. Rep..

[27] Lee K.Y., Mooney D.J. (2012). Alginate: Properties and biomedical applications. Prog. Polym. Sci..

[28] Vegas A.J., Veiseh O., Gürtler M., Millman J.R., Pagliuca F.W., Bader A.R., Doloff J.C., Li J., Chen M., Olejnik K. (2016). Long-term glycemic control using polymer-encapsulated human stem cell-derived beta cells in immune-competent mice. Nat. Med..

[29] Rinaudo M. (2006). Chitin and chitosan: Properties and applications. Prog. Polym. Sci..

[30] Francis Suh J.K., Matthew H.W.T. (2000). Application of chitosan-based polysaccharide biomaterials in cartilage tissue engineering: A review. Biomaterials.

[31] Wang Z., Zhang X., Gu J., Yang H., Nie J., Ma G. (2014). Electrodeposition of alginate/chitosan layer-by-layer composite coatings on titanium substrates. Carbohydr. Polym..

[32] Silva J.M., Duarte A.R.C., Custódio C.A., Sher P., Neto A.I., Pinho A., Fonseca J., Reis R.L., Mano J.F. (2014). Nanostructured Hollow Tubes Based on Chitosan and Alginate Multilayers. Adv. Healthc. Mater..

[33] Zhao Q., Han B., Wang Z., Gao C., Peng C., Shen J. (2007). Hollow chitosan-alginate multilayer microcapsules as drug delivery vehicle: Doxorubicin loading and in vitro and in vivo studies. Nanomed. Nanotechnol. Biol. Med..

[34] Martins G.V., Mano J.F., Alves N.M. (2010). Nanostructured self-assembled films containing chitosan fabricated at neutral pH. Carbohydr. Polym..

[35] Rúnarsson Ö.V., Malainer C., Holappa J., Sigurdsson S.T., Másson M. (2008). tert-Butyldimethylsilyl O-protected chitosan and chitooligosaccharides: Useful precursors for N-modifications in common organic solvents. Carbohydr. Res..

[36] Song W., Gaware V.S., Rúnarsson Ö.V., Másson M., Mano J.F. (2010). Functionalized superhydrophobic biomimetic chitosan-based films. Carbohydr. Polym..

[37] Sahariah P., Gaware V.S., Lieder R., Jónsdóttir S., Hjálmarsdóttir M.Á., Sigurjonsson O.E., Másson M. (2014). The Effect of Substituent, Degree of Acetylation and Positioning of the Cationic Charge on the Antibacterial Activity of Quaternary Chitosan Derivatives. Mar. Drugs.

[38] Signini R., Campana S.P. (1999). On the preparation and characterization of chitosan hydrochloride. Polym. Bull..

[39] Costa R.R., Custódio C.A., Arias F.J., Rodríguez-Cabello J.C., Mano J.F. (2011). Layer-by-Layer Assembly of Chitosan and Recombinant Biopolymers into Biomimetic Coatings with Multiple Stimuli-Responsive Properties. Small.

[40] Voinova M.V., Rodahl M., Jonson M., Kasemo B. (1999). Viscoelastic acoustic response of layered polymer films at fluid-solid interfaces: Continuum mechanics approach. Phys. Scr..

[41] Cerqueira M.T., Pirraco R.P., Santos T.C., Rodrigues D.B., Frias A.M., Martins A.R., Reis R.L., Marques A.P. (2013). Human adipose stem cells cell sheet constructs impact epidermal morphogenesis in full-thickness excisional wounds. Biomacromolecules.

[42] Poursamar S.A., Hatami J., Lehner A.N., da Silva C.L., Ferreira F.C., Antunes A.P.M. (2015). Gelatin porous scaffolds fabricated using a modified gas foaming technique: Characterisation and cytotoxicity assessment. Mater. Sci. Eng. C.

[43] Oliveira M.B., Custódio C.A., Gasperini L., Reis R.L., Mano J.F. (2016). Autonomous osteogenic differentiation of hASCs encapsulated in methacrylated gellan-gum hydrogels. Acta Biomater..

[44] Younes I., Rinaudo M. (2015). Chitin and chitosan preparation from marine sources. Structure, properties and applications. Mar. Drugs.

[45] Cho N.J., Kanazawa K.K., Glenn J.S., Frank C.W. (2007). Employing two different quartz crystal microbalance models to study changes in viscoelastic behavior upon transformation of lipid vesicles to a bilayer on a gold surface. Anal. Chem..

[46] Höök F., Kasemo B., Nylander T., Fant C., Sott K., Elwing H. (2001). Variations in coupled water, viscoelastic properties, and film thickness of a Mefp-1 protein film during adsorption and cross-linking: A quartz crystal microbalance with dissipation monitoring, ellipsometry, and surface plasmon resonance study. Anal. Chem..

[47] Lin S.J., Jee S.H., Hsaio W.C., Lee S.J., Young T.H. (2005). Formation of melanocyte spheroids on the chitosan-coated surface. Biomaterials.

[48] Chen Y.H., Wang I.J., Young T.H. (2009). Formation of keratocyte spheroids on chitosan-coated surface can maintain keratocyte phenotypes. Tissue Eng. A.

[49] Neto A.I., Vasconcelos N.L., Oliveira S.M., Ruiz-Molina D., Mano J.F. (2016). High-Throughput Topographic, Mechanical, and Biological Screening of Multilayer Films Containing Mussel-Inspired Biopolymers. Adv. Funct. Mater..

[50] Caridade S.G., Monge C., Almodóvar J., Guillot R., Lavaud J., Josserand V., Coll J.L., Mano J.F., Picart C. (2015). Myoconductive and osteoinductive free-standing polysaccharide membranes. Acta Biomater..

[51] Cuy J.L., Beckstead B.L., Brown C.D., Hoffman A.S., Giachelli C.M. (2003). Adhesive protein interactions with chitosan: Consequences for valve endothelial cell growth on tissue-engineering materials. J. Biomed. Mater. Res. A.

[52] Costa R.R., Alatorre-Meda M., Mano J.F. (2015). Drug nano-reservoirs synthesized using layer-by-layer technologies. Biotechnol. Adv..

